# Cost-effectiveness of a national exercise referral programme for primary care patients in Wales: results of a randomised controlled trial

**DOI:** 10.1186/1471-2458-13-1021

**Published:** 2013-10-29

**Authors:** Rhiannon Tudor Edwards, Pat Linck, Natalia Hounsome, Larry Raisanen, Nefyn Williams, Laurence Moore, Simon Murphy

**Affiliations:** 1 Centre for Health Economics & Medicines Evaluation, Institute of Medical and Social Care Research, Bangor University, Dean Street Building, Bangor LL57 1UT, UK; 2 DECIPHer, Cardiff School of Social Sciences, Cardiff University, 1-3 Museum Place, Cardiff CF10 3BD, UK; 3 North Wales Centre for Primary Care Research, Bangor University, College of Health and Behavioural Sciences, Brigantia Building, Bangor University, Bangor LL57 2UW, UK; 4 Centre for Primary Care and Public Health, Yvonne Carter Building, Queen Mary University of London, 58 Turner Street, London E1 2AB, UK

**Keywords:** Public health policy, Exercise referral, Mental health, Heart disease risk factors, Cost-effectiveness

## Abstract

**Background:**

A recent HTA review concluded that there was a need for RCTs of exercise referral schemes (ERS) for people with a medical diagnosis who might benefit from exercise. Overall, there is still uncertainty as to the cost-effectiveness of ERS. Evaluation of public health interventions places challenges on conventional health economics approaches. This economic evaluation of a national public health intervention addresses this issue of where ERS may be most cost effective through subgroup analysis, particularly important at a time of financial constraint.

**Method:**

This economic analysis included 798 individuals aged 16 and over (55% of the randomised controlled trial (RCT) sample) with coronary heart disease risk factors and/or mild to moderate anxiety, depression or stress. Individuals were referred by health professionals in a primary care setting to a 16 week national exercise referral scheme (NERS) delivered by qualified exercise professionals in local leisure centres in Wales, UK. Health-related quality of life, health care services use, costs per participant in NERS, and willingness to pay for NERS were measured at 6 and 12 months.

**Results:**

The base case analysis assumed a participation cost of £385 per person per year, with a mean difference in QALYs between the two groups of 0.027. The incremental cost-effectiveness ratio was £12,111 per QALY gained. Probabilistic sensitivity analysis demonstrated an 89% probability of NERS being cost-effective at a payer threshold of £30,000 per QALY. When participant payments of £1 and £2 per session were considered, the cost per QALY fell from £12,111 (base case) to £10,926 and £9,741, respectively. Participants with a mental health risk factor alone or in combination with a risk of chronic heart disease generated a lower ICER (£10,276) compared to participants at risk of chronic heart disease only (£13,060).

**Conclusions:**

Results of cost-effectiveness analyses suggest that NERS is cost saving in fully adherent participants. Though full adherence to NERS (62%) was higher for the economics sample than the main sample (44%), results still suggest that NERS can be cost-effective in Wales with respect to existing payer thresholds particularly for participants with mental health and CHD risk factors.

**Trial registration:**

Current Controlled Trials ISRCTN47680448

## Background

Lack of adequate physical exercise is a contributing factor in CHD risk, musculoskeletal problems, diabetes and depression which places a huge economic burden on the NHS and wider society in the UK [1] with similar trends worldwide. There is growing interest in general practice (GP) exercise referral schemes. Referred patients access an individually tailored exercise programme, including; exercise classes, swimming, green gyms and yoga.

Economic evaluation of public health interventions places challenges beyond the economic evaluation of clinical interventions in terms of potential wider benefits, multiple stake holders bearing costs, and time horizons over which costs and benefits will be accrued [2,3]. It is helpful to view such public health interventions as complex interventions with multiple components (in this case, fidelity to specific exercise interventions, compliance (dose) and uptake of the intervention, and mechanisms of behaviour change) [4,5].

Internationally, cost-effectiveness evidence has been equivocal compared with other population level interventions [6]. Matrix (2006), [7] reviewed the literature and modelled the cost-effectiveness of four interventions, pedometers, exercise referral, walking and cycling programmes in the community. Cost per QALY estimates for all four interventions were well below the NICE threshold of £20,000 to £30,000 per quality adjusted life year (QALY). Pavey et al. (2011), [8] showed a 51% probability of GP referral being cost-effective at £20,000 per QALY, 88% at £30,000 per QALY. In Australia, Cobiac et al. (2009), [6] modelled disability adjusted life years (DALYs) gained from six population level exercise promotion interventions. They found that pedometer use, mass media-based community campaigns, GP exercise referral, healthy transport and internet programmes were within the AUS $50,000 threshold.

There have been few prospective economic evaluations alongside pragmatic, community based RCTs of GP exercise referral schemes. Munro et al. (2004), [9] calculated a cost per QALY of €17,174 for a community-based exercise programme in over 65 year olds. Owen et al. (2011), [10] published a list of 200 public health interventions included in NICE guidance, of which exercise referral was one of the 85% of interventions under the £20,000 threshold. The above authors do not explore cost-effectiveness with respect to subgroups or explore how interventions address known inequalities in health, points recognised by Kelly et al. (2005) and Weatherly et al. (2009), [2,3]. In particular, how effective and cost effective ERS are for participants with a medical diagnosis which may benefit from exercise [8].

This economic evaluation was conducted alongside a pragmatic randomised controlled trial of the Welsh National Exercise Referral Scheme (NERS) [11,12]. Detailed effectiveness and headline economic results are published elsewhere [12]. NERS was designed to encourage increased activity in patients at low or medium risk of coronary heart disease or with mild to moderate depression. The scheme was sponsored by the Welsh Government (WG) in partnership with local authorities, National Public Health Service and Local Health Boards. The aim of our economic evaluation was to assess the cost-effectiveness of NERS in Wales from a multi-agency public sector perspective and to use subgroup analysis to explore the effects of medical diagnosis, gender, age, inequalities, referral route and adherence on effectiveness and cost-effectiveness.

## Methods

### Study design

A pragmatic, randomised controlled trial, with nested economic evaluation was conducted in 12 of the 22 local health board (LHB) areas in Wales, UK (for full details see study protocol) [11,12]. Participants aged >16 years, sedentary with risk factors for coronary heart disease (CHD) or minor mental health problems, were recruited by health professionals in primary care and randomised to intervention or control group. The intervention group participants were offered a 16-week exercise programme delivered by qualified exercise professionals (EP) based in local authority leisure centres. Participants allocated to the control group received an information leaflet and preferential access to the scheme after 12 months.

### Data collection and economic measures

Postal questionnaires were sent to all participants at baseline, 6 and 12 months. The questionnaire at 12 months included a willingness to pay question. Non-respondents received a reminder two weeks later. The Client Service Receipt Inventory [13] asked participants to recall their recent contacts with health care services in primary and secondary care, including prescribing. EQ-5D (3 L) [14] is a generic, preference based instrument for evaluating health-related quality of life. A minimal questionnaire pack was sent at baseline assessing demographics and activity levels to reduce participant burden and facilitate recruitment.

### Intervention costs

A breakdown of the budget for NERS for 13 local authorities initially providing NERS was obtained from WG. This budget included 50% of local authority exercise coordinators’ salaries, 100% of leisure centre exercise professionals’ (EP) salaries, training; promotional materials, administration, information technology, travel and equipment. Telephone interviews with NERS programme directors at WG and leisure centre managers were conducted to capture additional costs incurred. One local authority incurred costs, although it did not implement the scheme during the trial period.

### Cost and cost-effectiveness analysis

The mean national and local authority cost of running NERS for one year was used to calculate the range of costs and a cost per participant in the Scheme. We varied our assumptions around the number of referrals based on three estimates of participation;

a. referrals to the trial from July 2007 to October 2008, (n = 4779);

b. participants who accessed NERS during the trial, those in the intervention group (n = 1080) plus people referred to NERS, but not eligible for the trial (n = 1493), minus an estimated 15% who would not attend, (n = 2349).

c. and an arithmetic mean between our upper and lower estimates, (n = 3530).

The cost of the participants’ resource use was estimated in UK £, cost year 2008 using national sources [15-17].

Cost-utility analysis was conducted in line with MRC guidelines for the evaluation of complex interventions, [4] and our standard operating procedure for economic evaluation alongside pragmatic RCTs, [18]. When EQ-5D, [14] data was missing for one or two domains in the five domain scale, stochastic imputation (assuming data was missing at random) was used (n = 28 scores). Since health-related quality of life was assessed at 6 and 12 month follow-up only, the base-case analysis used area under the curve (AUC) data for the period between 6 and 12 months to estimate QALY change. Sensitivity analyses were conducted using: a) EQ-5D, [14] scores at 6 months for both intervention and control group as an estimate of baseline differences, and b) using control group mean EQ-5D scores at 6 months as a point estimate of EQ-5D, [14] score at baseline. Sensitivity analyses were conducted for: different intervention costs (£285, £385 and £579); referral to NERS by GP, nurse, or 50% referral by GP and 50% by nurse; participant payment of £1 per session, and of £2 per session (taking into account Wales Index of Multiple Deprivation; WIMD). Subgroup analyses compared the cost-effectiveness of NERS depending on gender, age group (<44, 44–60 or >60 years), adherence to the Scheme (16 weeks or <16 weeks), and referral reason (CHD and/or mental health risk factors). Cost-utility analyses were carried out using STATA SE 10. Costs and benefits were not discounted as the trial follow-up period was 12 months.

## Results

### Characteristics of economic sample

The economic sample, for whom full cost and EQ-5D, [14] data were collected (n = 798), represented 55% of the main effectiveness trial sample [12]. There were no statistically significant differences between the two samples with regard to gender of participants, or the WIMD [19]. However, the economic sample contained fewer younger participants (18%) than the effectiveness sample (30%), and a higher proportion of individuals who were referred because of CHD risk (77%) compared to the main sample (71%). There were no significant differences between the control and intervention groups in the economic sample with regard to demographic characteristics, levels of physical activity at baseline, referral reasons, and the mean number of reported visits to health professionals in the previous six months (Additional file 1: Table S1).

### The cost of the national exercise referral programme

Table 1 shows the total set up and annual operating costs of the NERS. Setup costs included salaries, meetings, printing and resources, translation costs and training for exercise professionals. Costs incurred by WG for the 6 pilot areas in 2006–07 are included as part of NERS development costs (£183,600). The estimated total setup costs incurred by the WG were £365,875; mean cost per local authority £28,144. Annual operating costs for NERS in 2007/08 was £1.36 million, mean per local authority was £104,602 (SD £27,226). Operating costs are inclusive of salaries for coordinators, exercise professionals, printing, administration, travel, staff management, additional training and room hire.

**Table 1:** **NERS set up costs for 13 local authorities in Wales in 2006 – 08 and operational costs for 2007 – 08 based on national budget plus national and local authority additional costs (£)**^**1**^

**2006-08**	**Total (£)**	**Mean (per LA)**	**S.D.**
**Set up**			
***National costs***			
*Salaries*			
*Physical activity specialist* (*0*.*2 WTE in yr 1*, *0*.*6 WTE yr 2*)	*39*,*317*		
*Line Management* (*0*.*02 WTE yrs 1&2 Grade 7*)	*5*,*823*		
*Executive officer* (*0*.*2 WTE yr 2*)	*4*,*254*		
Exercise professionals training (Level 3)	51,759		
Meetings	3,852		
Resources & printing	9,845		
Translation costs	537		
Pilot exercise referral project	183,587		
***Additional Local Authority costs***			
*IT*	*43*,*004*	*3*,*308*	*1*,*775*
*Equipment*	*15*,*290*	*1*,*176*	*874*
*Staff clothing*	*1*,*196*	*92*	*218*
*Attending meetings*	*4*,*030*	*310*	*506*
*Promotion or advertising*	*2*,*210*	*170*	*316*
*Home working facilities*	*1*,*170*	*90*	*246*
***Total set up***	***365,*** ***875***	***28,*** ***144***	***2,*** ***475***
**Annual operational costs 2007-08**			
***National costs***			
WAG salaries			
Physical activity specialist (0.8)	39,298		
Line Management (0.02 WTE Grade 7)	3,515		
Executive officers (1.2 WTE)	26,618		
Meeting costs	2,855		
National resources	20,324		
Exercise professionals (36 WTE)	781,833	60,141	24,213
Printing, stationary and administration	19,750	1,646	829
Training	18,967	1,459	916
Travel	47,351	3,642	1,496
***Joint national and local costs***			
Co-ordinator salary and on-costs (13 WTE 50% funded by WAG & LA)	358,574	27,583	3,995
***Local authority costs***			
Staff management	29,250	2,250	n/a
Promotional material	715	55	151
Room hire	10,326	794	1,453
Attending conferences	455	35	96
**Total NERS annual operational cost**	**£1,359,832**	**104,602**	**27,226**
**Cost per participant in NERS**	N or £		
Participants in NERS (N)^2^	2,349		
**Participant cost in 16 week programme based on intervention group**^**3**^	**£579**		
All referrals July 2007 – October 2008 (12 months in study period) (N)	4,779		
**Participant cost in 16 week programme**^**3**^	**£285**		
Arithmetic mid-point between referrals and participants	3,530		
**Base case intervention per person cost using N above**	**£385**		
Participants in the economic analysis who supplied service utilisation data	798		

^1^All costs in UK£ rounded to nearest whole £.

^2^People who accessed NERS during the trial, those in the intervention group (n=1080) plus the people referred who were eligible for NERS but not for the trial, minus 15% non-attenders (n=1493), a total of 2349.

^3^Calculation - total annual operational cost divided by N.

On an intention-to-treat basis, to estimate the cost per participant in NERS, we divided the total annual operating costs for NERS for 2007/08 (£1,359,832), by 52 (weeks), multiplied by 16 (16 week programme), then divided this figure by the three estimates of number of referrals to NERS across the participating local authorities. We report a base case ICER calculation using the mid point estimate of the cost per participant (£385, n = 3530), in addition to an upper (£579, n = 2349) and lower estimate (£285, n = 4779), (Table 1).

### Opportunity costs

Leisure centre managers reported no lost revenues from providing NERS, as sessions were held at quieter times of the day, thus utilizing underused space.

### Use of health services

National unit costs of health services used in the calculations can be accessed as Additional file 2: Table S2. Table 2 shows the frequency and total mean costs of service use by intervention and control groups, subdivided into primary care (including prescribing) and secondary care. For the 12 month study period there was no significant difference in NHS resource use between the intervention and control groups, apart from the costs of healthcare tests, higher for the control group (p = < 0.05).

**Table 2:** NERS resource use and costs over 12 months by group

	**Intervention group**	**Control group**	**Significance**^**1**^
	**n = 400**	**n = 398**	***P***
**Primary Care Sector**	*Mean*, *median* (*min*, *max*)		
GP consultations, surgery	5.18, 4 (0, 28)	6.00, 5 (0, 46)	0.11
Phone	0.63, 0 (0, 14)	0.69, 0 (0, 20)	0.68
Home visits	0.12, 0 (0, 20)	0.11, 0 (0, 10)	0.64
Practice nurse consultations	2.98, 1 (0, 29)	3.31, 2 (0, 42)	0.05
Mental Health Professionals	0.83, 0 (0, 32)	0.71, 0 (0, 53)	0.58
Other health professionals	0.65, 0 (0, 32)	1.14, 0 (0, 36)	0.11
**Secondary Care Sector**			
Consultant	2.00, 1 (0, 22)	2.09, 1 (0, 26)	0.55
Specialist nurse	0.87, 0 (0, 42)	0.61, 0 (0, 24)	0.98
Physiotherapist	1.59, 0 (0, 52)	1.66, 0 (0, 48)	0.63
Other health professionals seen	0.68, 0 (0, 78)	0.35, 0 (0, 26)	0.21
A&E	0.32, 0 (0, 18)	0.30, 0 (0, 15)	0.08
Inpatient hospital days (all causes)	0.97, 0 (0, 96)	1.05, 0 (0, 32)	0.70
**Type of Cost**^**2**^	**Intervention**	**Control group**	**Mean difference**
	**Mean (SD)**	**Mean (SD)**	**(95% CI bootstrapped)**
**NHS primary care sector**			
**GP consultations:**			
Surgery	186 (159)	215 (204)	-28
Telephone	14 (36)	15 (40)	-1.4
Home visits	6 (55)	6 (35)	0.7
*All GP consultation* (*subtotal*)	*206* (*182*)	*235* (*224*)	-*29* (-*59*, -*0*.*3*)
Practice nurse consultations	51 (104)	56 (125)	-5
Mental health professional	42 (189)	36 (198)	6
Other health professionals	23 (87)	31 (121)	-9
***Total primary care consultations***	***322 (339)***	***358 (409)***	***-36.*** ***5 (-92, 16)***
Primary care prescribing	352 (448)	391 (494)	-39
**Total primary care**	**674 (610)**	**749 (712)**	**-76 (-167, 14)**
**NHS secondary sector**			
**Outpatients:**			
Consultant	223 (302)	232 (325)	-9 (-51, 33)
Specialist nurse	312 (127)	21 (69)	11 (-2, 24)
Physiotherapist	65 (223)	68 (202)	-3 (-31, 26)
Other hospital attendances	60 (598)	17 (107)	43 (-3,110)
Day cases	121 (361)	83 (330)	38 (-12, 89)
Inpatient hospital days (all causes)	374 (1915)	411 (1447)	-37 (-246, 210)
A&E attendances	26 (107)	23 (78)	2 (-10, 16)
Tests	121 (251)	149 (258)	-28 (-64, 7)*
**Total secondary care**	**901 (2235)**	**856 (1721)**	**45 (-208, 351)**
**TOTAL NHS COST**	**1695 (2514)**	**1754 (2155)**	**-58.4 (-361, 301)**
**Base case NERS Intervention cost**			
2 session/week for 16 week	385	0 (0)	385
**TOTAL NHS, WAG and LOCAL AUTHORITY COST**	**2080 (2514)**	**1754 (2155)**	**326 (-2, 660)**

^1^Mann–Whitney test (exact two-sided significance level) No significant differences found.

^2^Costs rounded to nearest £.

*The difference is significant (P < 0.05) according to Mann–Whitney test.

### Adherence to NERS

Approximately 62% completed the 16 week programme, 32% (n = 123) attended fewer than 16 weeks, and 8% (n = 30) did not attend at all. Table 3 breaks down adherence by reason for referral, gender, age and the WIMD, [19]. Those at risk of CHD were more likely to adhere to the full programme than those with mental health problems, and those with both mental health problems and at risk of CHD. A higher percentage of men completed the programme than women. Those people living in areas of high deprivation were more likely to complete the programme (66%) compared with those living in areas of low deprivation (60%).

**Table 3:** Mean effectiveness outcomes by group over 12 months

**Outcomes at 12 month follow-up**	**Intervention group**		**Control group**	Significance*
	**Mean (SD)**		**Mean (SD)**	***p***
**EQ5D score (0-1)	0.64 (0.32) n=395		0.61 (0.32) n=391	0.09
Exercise per week	305.96 min (386.5) n=298		305.8 min (393.3) n=279	0.7
**Adherence**				
Did not attend	30 (8%)			
Attended <16 wks	123 (32%)			
Attended 16 wks	247 (62%)			
**Level of adherence to NERS by variable**		**Did not attend**	**0-16 week**	**Completed**
		**N (%)**	**N %)**	**N (%)**
Referral type	CHD risk	20 (7)	84 (27)	203 (66)
	MH	0	4 (31)	9 (69)
	Both	10 (12)	35 (44)	35 (44)
Gender	Male	15 (11)	31 (33)	89 (66)
	Female	15 (5)	92 (35)	158 (60)
Age	<44	5 (7)	25 (36)	39 (57)
	45-59	10 (7)	50 (27)	75 (56)
	>60	15 (8)	48 (24)	133 (68)
WIMD	Low	7 (5)	49 (35)	84 (60)
	Medium	13 (10)	37 (28)	84 (63)
	High	9 (8)	29 (26)	73 (66)

*The difference is significant (*p* < 0.05) according to Mann–Whitney test.

**EQ-5D (L3).

### Participants’ willingness to pay for exercise classes

At 12 month follow-up, 55% completed the willingness to pay question (n = 441). The amount people were willing to pay was £2.16 (SD £1.50) for those in the intervention group compared with £2.38 (SD £1.78) in the control. This had fallen from the baseline figure of £2.43 (SD £1.67) compared with £2.55 (SD £2.49) in the control (81% response, n = 648). There was a difference in willingness to pay (WTP) depending upon whether people lived in an area of low or high deprivation as measured by the WIMD. At 12 month follow-up people from areas of high deprivation were WTP £1.91 (SD £0.88) compared with £2.05 for those from areas of low deprivation. Similarly, in the control group people were willing to pay £2.09 (SD £1.53) and £2.32 (SD £1.78), respectively. In all groups people valued the intervention more highly if they were in the control group. When considering the WTP by employment status then students were willing to pay the most per session and those seeking work the least.

### Cost-utility analysis

**Figure 1:**
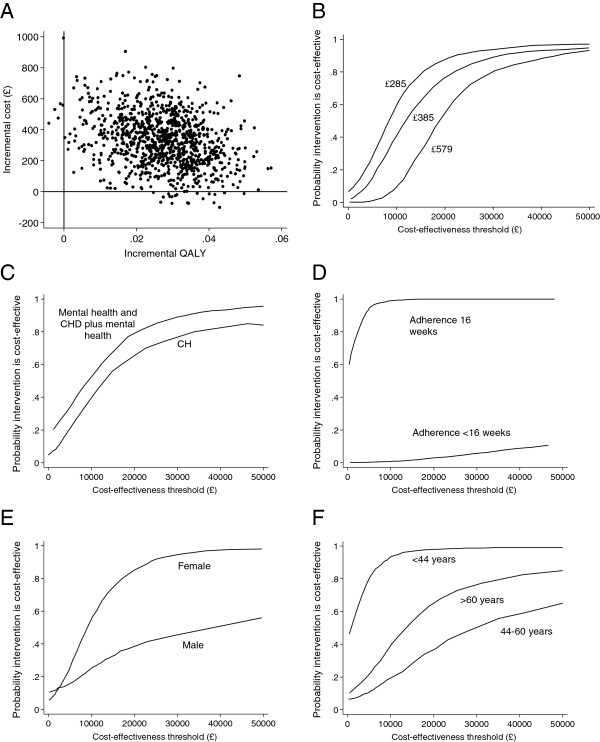
**Cost-effectiveness planes with 1,000 bootstrapped ICER estimates for NERS economic evaluation.** Cost-effectiveness planes for base case **(A)**, cost-effectiveness acceptability curves for three intervention costs of £285, £385 (base case) and £579 **(B)**, and for participant subgroups with different referral reason to NERS **(C)**, adherence to NERS **(D)**, gender **(E)**, and age of participants **(F)**.

The base case analysis produced a mean difference in cost of £327 and a mean difference in QALY gains of 0.027 between groups, indicating NERS was more expensive and more effective compared to the control. The incremental cost-effectiveness ratio (ICER) was £12,111 per QALY gained. Figure 1 shows the cost-effectiveness plane with 1,000 bootstrapped ICER estimates. The figure demonstrates that the majority of simulations fell in the north-east quadrant of the cost-effectiveness plane, where the intervention is more expensive and more effective compared to the control condition.

### Sensitivity analyses

Probabilistic sensitivity analysis conducted for our base case demonstrated that there is a 77% probability of NERS being cost-effective at £20,000 per QALY and 89% at £30,000 per QALY gained (Table 4). To assess the uncertainty associated with cost of NERS, sensitivity analyses were performed assuming an annual cost of attending the programme of either £285 or £579 per person per year. The ICER point estimates were £8,556 and £19,296 per QALY, respectively, remaining below a £30,000 payer threshold. However, for an annual cost of attending the NERS programme of £579 per person, the probability of the scheme being cost-effective was lower than the base case at 54% and 70% at £20,000 and £30,000 thresholds, respectively.

**Table 4:** Results of cost-utility analysis

	**Incremental cost (£)**	**Incremental QALY**	**ICER point estimate (£) (Bootstrapped one sided 95% CI for ICER (£))**	**Probability intervention is cost-effective at £20,000 per QALY (%)**	**Probability intervention is cost-effective at £30,000 per QALY (%)**
***Base case***					
Cost of intervention £385	327	0.027	12,111 (58,881)	77	89
***Sensitivity analysis***					
EQ-5D at 6 months as estimate of baseline	327	0.054	6,056 (37,159)	93	96
Control group mean EQ-5D at 6 months as estimate of baseline	327	0.046	7,109 (24,853)	95	98
Participant payment of £1 per session	295	0.027	10,926 (69,085)	80	91
Participant payment of £2 per session	263	0.027	9,741 (64,638)	83	92
***Subgroup Analysis***					
**Referral reason**	239	0.0183	13,060 (117,893)	65	76
CHD	596	0.058	10,276 (50,925)	80	89
Mental health and CHD plus mental health					
**Gender**					
Male	322	0.0084	38,333 (254,973)	39	47
Female	326	0.0362	9,006 (39,000)	85	95
**Age**					
<44	68	0.0656	1,037 (16,418)	98	99
44-60	577	0.0179	32,235 (314,108)	37	51
>60	244	0.0187	13,048 (153,565)	67	76
**Adherence to NERS**					
<16 weeks	662	-0.0084	Dominated	2.9	5.6
16 weeks	-18	0.049	Dominant	100	100

N/A, not applicable.

When EQ-5D, [14] data at 6 months was used as an estimate of baseline EQ-5D scores, the difference in QALY gain between the intervention and control groups was 0.054, resulting in a decrease in ICER estimate from £12,111 to £6,056. Using the control group mean EQ-5D, [14] score at 6 months as an estimate of baseline values for both intervention and control groups, produced a QALY difference of 0.046 and an ICER point estimate of £7,109. Probabilistic sensitivity analyses demonstrated that alternative methods of QALY calculation resulted in higher probabilities of NERS being cost-effective compared to our base case, which can be considered conservative (Table 4).

Pragmatic sensitivity analyses were conducted to compare referrals to NERS by a GP or a nurse. When all patients were referred by a GP this resulted in a higher ICER (£13,444) compared to referral by nurse (£12,667) or 50-50% by GP and nurse (£13,074) (probability of being cost-effective was 72%,76% and 75% respectively at £20,000 threshold; and 87%, 89% and 88% respectively at £30,000 threshold). When participant payments of £1 and £2 per session were considered in the cost-utility analysis, the cost per QALY fell from £12,111 to £10,926 and £9,741, respectively. The probability of NERS being cost-effective at £30,000 per QALY increased from 89% to 91% and 92%, with the £1 and £2 per session participants’ contributions (Table 4).

### Subgroup analyses

Since the participant sample used in our cost-utility analysis was heterogeneous with relation to age (16–88 years), gender, reason for referral and adherence to NERS, a range of subgroup analyses were conducted to identify population groups which would most benefit from joining the NERS Scheme (Table 4). The cost-effectiveness acceptability curves (CEACs), (Figure 1C-F) demonstrated NERS is more likely to be cost-effective in younger participants (<44 years) than in older individuals. Participants with a mental health risk factor alone or in combination with a risk of chronic heart disease were characterized by a lower ICER (£10,276) compared to participants at risk of chronic heart disease only (£13,060). Subgroup analysis by gender, gave a significantly lower ICER for women (£9,006) than for men (£38,333). Pronounced differences in ICER were found between fully adherent participants and those attending fewer than 16 sessions (Table 4). For participants adhering to NERS, the analysis resulted in a net saving of £18 per patient per year. The probability that NERS is cost-effective in adherent participants was 100% at £20,000 (Figure 1).

## Discussion

The main trial effectiveness findings [12] demonstrated that the NERS was effective in increasing weekly exercise and improved self-reported mental health at 12 months as compared to the control group. A larger effect was found for women versus men and for persons who completed the 16 week programme, as compared with those who were non-adherent. Results of cost-effectiveness analyses suggest that NERS is likely to be cost saving in fully adherent participants [8]. Our base case analysis and sensitivity analyses, we see that the ICER for NERS under different assumptions falls largely below a payer threshold of £20-30,000. However we recognize the need for caution in interpretation of ICERs which have an upper 95% confidence limit above £20,000 (Table 4). From a methodological viewpoint, the NERS trial shows the benefits of incorporating subgroup analysis and analysis of how public health interventions may impact on inequalities in health through i.e. differing uptake and effects by women and men, and differing willingness to pay by areas of socioeconomic deprivation. Our conservative base case analysis is robust to a range of sensitivity analyses, leading to the conclusion that NERS is likely to be cost-effective, under the £20,000 to £30,000 threshold (89% probability at £30,000).

Decisions based on average measures of cost-effectiveness carry a risk of overlooking potential benefits for a given subset of the population. In the current climate of limited health care resources, there is a clear necessity to maximise population health by delivering healthcare interventions to those recipients who would most benefit, [19]. The proposed ways to account for heterogenity between population groups included: reporting ICER estimates for different subgroups, incorporating covariate adjustment in cost-effectiveness analysis, and using alternative cost-effectiveness indicators, [20-24]. Our analysis revealed wide variations in ICERs between subgroups.

Our analysis shows that adherence to exercise was greater in those areas of high deprivation as measured by the WIMD, [19]. We also found that those people living in areas of high deprivation placed a higher value on NERS than those living in areas of lower socioeconomic deprivation. Our study goes some way to address current thinking in public health economics about the need to consider equity implications of access and uptake, in addition to cost-effectiveness, [10].

### Comparison with other literature

Economic evaluation of exercise referral schemes involving inactive adults in primary care have been conducted as a part of the NIHR Health Technology Assessment programme, [12]. The base-case cost-effectiveness analysis of NERS in Wales generated comparable data with previous reports, [6-9,25] demonstrating that the exercise referral schemes may be cost-effective under the £30,000 per QALY threshold. However, lifetime modelling exercises indicate a greater uncertainty of the long term cost-effectiveness of such programmes, [26]. Our study suggests that NERS would be also be cost-effective in individuals at risk of CHD and mental health issues, as well as in individuals aged 60 years and over. The NERS may be cost-saving assuming full adherence to the programme.

### Strengths and limitations

There have been few rigorous prospective economic studies alongside RCTs of exercise referral interventions. Results of the NERS trial, as a pragmatic policy evaluation, are likely to have high external validity and generalisabilty, [27]. Full details of the methodological design of the NERS trial and its acknowledged limitations are published elsewhere, [11,12]. The NERS trial set out to explore the potential effectiveness and cost effectiveness of a national exercise referral scheme, integrating motivational interviewing, and making use of existing leisure centres. The NERS trial took a pragmatic approach, in that it was powered to show a difference in reported exercise at 12 months, by telephone, using 7D-PAR, a well validated measure, rather than focussing on load i.e. exercise type, or intensity. The NERS trial did focus on patients with CHD risk factors, mental health problems or both, but did not specify exercise type. Indeed the exercise was tailored, and this tailoring may explain the relatively high adherence rate to the programme. The NERS trial differs from published trials of specific exercise programmes for people with specific diagnoses, and hence economic evaluation of the NERS trial in terms of cost-utility analysis differs from cost-utility analyses of specific exercise programmes for people with specific diagnoses, [28]. The trial specifically recruited patients with CHD risk factors, mental health problems or both conditions. The trial found that NERS was effective in increasing physical activity among those referred with CHD risk factors. Although there was no increase in physical activity among those referred for mental health reasons, anxiety and depression were reduced. These effects were highly dependent on adherence to the programme. To these results [12], this paper adds cost per QALY estimates for NERS, for comparison with payer thresholds, such as that of NICE, and for comparison with those of other large, community based ERS, [25]. The NERS trial allowed a fully integrated economic evaluation which took a wide perspective, measuring costs and benefits to the WG, local authority leisure centres as well as the NHS. To reiterate, the methodological design of the NERS trial was more about achieving external validity in terms of policy evaluation at a macro level, than about micro level evaluation of a specific exercise programme for a specific diagnosis or patient group, in a sports science context.

A limitation of our study was that baseline EQ-5D, [14] data was not available. However, on the small set of baseline measures which were taken, activity level, service use over the preceding 6 months, age and gender, the intervention and control groups were similar. Our economic analysis was based on a smaller number of participants, because not all participants completed the economic questionnaires. We acknowledge that using a smaller number of participants in subgroup analysis increases statistical uncertainty. Reported ICER point estimates have a rather explorative value and require further verification.

We cannot extrapolate beyond the 12 month follow up period of the trial which is a limitation common to many trials of public health interventions. A future modeling exercise may help address the question of whether such an exercise intervention could have longer term benefits.

The effectiveness and economic results of the NERS trial suggest that age, gender and adherence all have an important part to play in affecting weekly exercise behaviour and self reported, health related quality of life. Weatherly et al. (2009), [3] have stressed the need for economic evaluations of public health interventions to examine equity considerations. Our results indicate that people living in areas of higher deprivation who were offered NERS were likely to adhere to the complete programme, although more research would be needed to investigate if this was maintained in the long-term.

## Conclusions

Though full adherence to NERS (62%) was higher for the economics sample than the main sample (44%), our base case analysis over a 12 month follow up period, is robust to a range of sensitivity analyses. ICERs were well below the NICE threshold of £20-30,000, though upper 95% confidence limits cross this boundary, indicating the need for caution in the interpretation of results. There is evidence for confidence that NERS is likely to be cost saving in fully adherent participants, leading to the overall conclusion that NERS can be cost-effective.

## Competing interests

The authors declare that they have no competing interests.

## Authors’ contributions

RTE was responsible for design of the economic evaluation and overseeing the economic evaluation. PL was responsible for the day-to-day running of the economic evaluation. NH was responsible for the cost-effectiveness analysis. RTE, PL and NH produced a first draft of the manuscript. LR was trial manager, SM was principal investigator. LM and NW were part of the trial management team. All authors read and commented on drafts and approved the final manuscript and had full access to all the data (including statistical reports and tables) in the study and can take responsibility for the integrity of the data and the accuracy of the data analysis. RTE acts as guarantor. All authors read and approved the final manuscript.

## Pre-publication history

The pre-publication history for this paper can be accessed here:

http://www.biomedcentral.com/1471-2458/13/1021/prepub

## Supplementary Material

Additional file 1: Table S1 Baseline characteristics by group (values are number and percentages unless otherwise stated). Click here for file

Additional file 2: Table S2 Unit cost of health service use costs in UK pounds for 2007–08 (£) with source of costs^a^. Click here for file
