# Supplementary material for: Cost-effectiveness of a national exercise referral programme for primary care patients in Wales: results of a randomised controlled trial
Source: BMC Public Health. 2013 Oct 29;13:1021. doi: 10.1186/1471-2458-13-1021 (PMC4231449; doi:10.1186/1471-2458-13-1021)
Supplement: Additional file 1: Table S1 — Baseline characteristics by group (values are number and percentages unless otherwise stated). [file 1471-2458-13-1021-S1.doc]

**ADDITIONAL FILE 1: TABLE S1**

**Title:** Supplementary Table 1: Baseline characteristics by group

**Legend:** Supplementary Table 1: Baseline characteristics by group (values are number and percentages unless otherwise stated)

|  | **Intervention group**  **n=400** | **Control group**  **n=398** |
| --- | --- | --- |
| **Gender** |  |  |
| Male | 135 (34%) | 130 (33%) |
| Female | 265 (66%) | 268 (67%) |
| **Age** |  |  |
| Mean, (SD), *min ,max,* | 57.3, (13.5),*16,85,* | 56.7, (13.2) *17,88* |
| <44 years | 69 (17%) | 71(18%) |
| 45-59 years | 135 (34%) | 136 (34%) |
| >60 years | 196 (49%) | 191 (48%) |
| **Employment** |  |  |
| In employment | 105 (26%) | 98 (25%) |
| Retired | 165 (41%) | 166 (42%) |
| Housework | 65 (16%) | 75 (19%) |
| In education | 7 (2%) | 4 (1%) |
| Seeking work | 14 (4%) | 9 (2%) |
| Other | 41 (10%) | 38 (9%) |
| Missing | 3 (1%) | 8 (2%) |
| **Wales Index of Multiple Deprivation** |  |  |
| Low | 140 (35%) | 128(32%) |
| Medium | 134(34%) | 152(38%) |
| High | 111(28%) | 111(28%) |
| Missing | 15 (4%) | 7(2%) |
| **Physical activity** |  |  |
| Inactive | 225 (56%) | 242 (61%) |
| Moderately inactive | 59(15%) | 54 (5%) |
| Moderately active | 71 (18%) | 58 (15%) |
| Active | 34(9%) | 39 (10%) |
| Missing | 11 (3%) | 5(1%) |
| **Number of visits to health professionals** |  |  |
| Mean, (SD) *median, min, max* | 6.46 (8.2), *4, 0, 65* | 6.29,(10.4) *4, 0, 117* |
| **Reason for referral to NERS** |  |  |
| At risk of CHD | 307 (77%) | 309 (78%) |
| Mild-moderate mental health problem | 13 (3%) | 13 (3%) |
| Both | 80 (20%) | 76 (19%) |
