# Supplementary material for: Cost-effectiveness of a national exercise referral programme for primary care patients in Wales: results of a randomised controlled trial
Source: BMC Public Health. 2013 Oct 29;13:1021. doi: 10.1186/1471-2458-13-1021 (PMC4231449; doi:10.1186/1471-2458-13-1021)
Supplement: Additional file 2: Table S2 — Unit cost of health service use costs in UK pounds for 2007–08 (£) with source of costsa. [file 1471-2458-13-1021-S2.doc]

**ADDITIONAL FILE 2: TABLE S2**

**Title:** Supplementary Table 2: Unit costs of health service use in UK pounds 2007-08

**Legend:** Supplementary Table 2: Unit cost of health service use costs in UK pounds for 2007-08 (£) with source of costs^a^

| **Healthcare resource** | **Unit** | | **Unit cost** | **Details** |
| --- | --- | --- | --- | --- |
| GP (surgery) | Visit | | 36 | Per 11.7 mins consultation ^9^ |
| GP (telephone) | Consultation | | 22 | Per 23.4 mins. consultation ^9^ |
| GP (home visit) | Visit | | 58 | Per 23.4 mins consultation Incl.travel ^9^ |
| GP clinic | Visit | | 52 | Per 17.2 mins consultation Incl. of travel ^9^ |
| GP out of hours service |  | | 36 | Costed as GP in surgery |
| Specialist nurse (Surgery) | Visit | | 15 | Per 15 mins consultation ^9^ |
| Specialist nurse (Phone) | Visit | | 8 | Per 6 mins. Consultation (Based on above) |
| Community/specialist nurse (Home Visit) | Visit | | 39 | Per 20 mins consultation. ^9^ |
| Community mental health nurse | Hour | | 51 | Per contact hour ^9^ |
| Psychologist | Hour | | 72 | Per client contact hr plus travel ^9^ |
| Other health professional seen, eg. counsellor, physio, chiropodist etc. | Visit | | Various | Per clinic or home consultation ^9^ |
| Prescribing | Item | | Various | Per item cited by BNF ^11^ |
| Tests and Investigations | Procedure | | Various | Direct access pathology and diagnostic, ^10^ |
| Secondary care |  | |  |  |
| Consultant out patients | Consultation | | Various | Costed by speciality ^10^ |
| Specialist nurses and other health professionals | Consultation | | Various | Costed by speciality ^10^ |
| A&E | Consultation | | Various | Depending on severity and admission ^10^ |
| Day surgery | Procedure | | Various | Costed by treatment ^10^ |
| In-patient | Procedure | | Various | Costed by treatment for average length of stay^10^ |
| In patients, excess stay | Day | | Various | Costed by treatment for excess length of stay^10^ |
| **Base case NERS intervention cost** | | | | |
| 16 week exercise programme, 2 session a week | |  | £385 | Mean cost per participant based on information from WAG and local authorities |

**^a^** NHS costs include salary, on-costs, qualifications overheads and capital costs all rounded to nearest whole £.
